# Supplementary material for: Investigating Performance in a Strenuous Physical Task from the Perspective of Self-Control
Source: Brain Sci. 2019 Nov 9;9(11):317. doi: 10.3390/brainsci9110317 (PMC6896147; doi:10.3390/brainsci9110317)
Supplement: Supplementary file 1 [file brainsci-09-00317-s001.pdf]

Supplementary Materials: Study Materials

Ablaufplan erster Termin: Studie zur Kraftleistung

Bitte lesen Sie die folgenden Informationen aufmerksam durch. Wenn Ihnen etwas unklar ist, dann fragen Sie bitte die Versuchsleitung.

Die Bezahlung

In der Kraftleistungsaufgabe können Sie Geld verdienen: Jeder Durchgang dauert eine Minute. **Für jeden Durchgang bekommen Sie einen Euro. Sie bekommen also einen Euro pro Minute.** Wenn Sie 10 komplette und einen halben Durchgang machen, dann bekommen Sie 10 Euro und 50 Cent, da Sie die Kraftleistungsaufgabe dann 10 Minuten und 30 Sekunden ausgeführt haben. Entscheiden Sie selbst, wie lange Sie die Kraftleistungsaufgabe machen wollen. Es werden nur korrekt durchgeführte Durchgänge vergütet. Lesen Sie daher bitte aufmerksam durch, wie die Kraftleistungsaufgabe durchgeführt wird und was die Abbruchkriterien sind.

Die Kraftleistungsaufgabe

In der Kraftleistungsaufgabe geht es darum, mit Ihrem Oberschenkel (Quadrizeps), dauerhaft eine bestimmte Kraftleistung zu erbringen: Die rote Linie stellt „live" Ihre erbrachte Kraftleistung dar und die schwarze horizontale Linie gibt an, wie viel Leistung zu erbringen ist. Korrekt ausgeführt ist die Kraftleistungsaufgabe dann, wenn die rote Linie nie die schwarze horizontale Linie unterschreitet.

In jedem Durchgang werden wir Sie nach 48 Sekunden fragen, wie **anstrengend** die Aufgabe ist. Nach 52 Sekunden werden wir Sie fragen, wie stark Sie **Schmerz** empfinden. Die Skalen mit denen wir Anstrengung und Schmerz erfassen sind links von Ihnen an der Wand angebracht. Bitte prägen Sie sich vorab die Bedeutung der Werte ein und nennen Sie uns dann immer nur die für Sie in diesem Moment zutreffende Zahl (z.B. auf die Frage **„Anstrengung?"**, sagen Sie **„3"**, wenn Sie die Kraftleistungsaufgabe in diesem Moment als **„Moderat"** anstrengend empfinden).

Nach 57 Sekunden entspannen Sie sich für drei Sekunden vollständig. Hier informieren wir Sie, wieviel Geld Sie aktuell verdient haben und wie viel Sie verdienen würden, wenn Sie sich entscheiden einen weiteren Durchgang zu machen. Danach beginnt automatisch der nächste Durchgang. Machen Sie so viele Durchgänge wie Sie möchten.

Wenn Sie aufhören wollen, dann sagen Sie bitte deutlich vernehmbar **„Stop"** und entspannen sich komplett. Nach einigen Sekunden werden wir Sie ermuntern, noch einmal Ihre maximale Kraftleistung zu erbringen (wie eben im warm-up). Im Anschluss können Sie sich dann komplett entspannen. Hier werden wir Sie noch zweimal stimulieren: Wenn Sie ihre maximale Leistung erbringen und im Anschluss, wenn Sie sich komplett entspannen.

Abbruchkriterien

- Wenn die rote Linie in einem Durchgang dreimal, für maximal eine Sekunde, unter die schwarze horizontale Linie geht, dann wird der Test abgebrochen.
- Wenn die rote Linie in einem Durchgang einmal, für länger als eine Sekunde, unter die schwarze horizontale Linie geht, dann wird der Test ebenfalls abgebrochen.

Zusammenfassung

Sie bearbeiten die Kraftleistungsaufgabe **so lange Sie möchten**. Für jeden Durchgang bekommen Sie einen Euro. Sie bekommen also **einen Euro pro Minute**. Die Messung geht so lang, **bis Sie aufhören wollen** oder **eines der Abbruchkriterien eintritt**.
Wichtig**: Wenn Sie aufhören wollen, dann sagen Sie bitte deutlich vernehmbar „Stop"**.

Haben Sie noch Fragen? Ansonsten kann es losgehen!

A.8. Study Procedure Session 2- ICWD Scenario

Ablaufplan zweiter Termin: Studie zur Kraftleistung

Bitte lesen Sie die folgenden Informationen aufmerksam durch. Wenn Ihnen etwas unklar ist, dann fragen Sie bitte die Versuchsleitung.

Die Bezahlung

In der Kraftleistungsaufgabe können Sie Geld verdienen: **Heute bekommen Sie für jeden Durchgang 50 Cent.** Wenn Sie 10 komplette und einen halben Durchgang machen, dann bekommen Sie also 5 Euro und 25 Cent, da Sie die Kraftleistungsaufgabe dann 10 Minuten und 30 Sekunden ausgeführt haben **Da die Bezahlung pro Durchgang heute nur halb so hoch ist, erhalten Sie vorab einen Bonus von XX Euro.** Entscheiden Sie selbst, wie lange Sie die Kraftleistungsaufgabe machen wollen. Es werden nur korrekt durchgeführte Durchgänge vergütet. Lesen Sie daher bitte aufmerksam durch, wie die Kraftleistungsaufgabe durchgeführt wird und was die Abbruchkriterien sind.

Die Kraftleistungsaufgabe

In der Kraftleistungsaufgabe geht es darum, mit Ihrem Oberschenkel (Quadrizeps), dauerhaft eine bestimmte Kraftleistung zu erbringen: Die rote Linie stellt „live“ Ihre erbrachte Kraftleistung dar und die schwarze horizontale Linie gibt an, wie viel Leistung zu erbringen ist. Korrekt ausgeführt ist die Kraftleistungsaufgabe dann, wenn die rote Linie nie die schwarze horizontale Linie unterschreitet.

In jedem Durchgang werden wir Sie nach 48 Sekunden fragen, wie **anstrengend** die Aufgabe ist. Nach 52 Sekunden werden wir Sie fragen, wie stark Sie **Schmerz** empfinden. Die Skalen mit denen wir Anstrengung und Schmerz erfassen sind links von Ihnen an der Wand angebracht. Bitte prägen Sie sich vorab die Bedeutung der Werte ein und nennen Sie uns dann immer nur die für Sie in diesem Moment zutreffende Zahl (z.B. auf die Frage **„Anstrengung?“**, sagen Sie **„3“**, wenn Sie die Kraftleistungsaufgabe in diesem Moment als **„Moderat“** anstrengend empfinden).

Nach 57 Sekunden entspannen Sie sich für drei Sekunden vollständig. Hier informieren wir Sie, wieviel Geld Sie aktuell verdient haben und wie viel Sie verdienen würden, wenn Sie sich entscheiden einen weiteren Durchgang zu machen. Danach beginnt automatisch der nächste Durchgang. Machen Sie so viele Durchgänge wie Sie möchten.

Wenn Sie aufhören wollen, dann sagen Sie bitte deutlich vernehmbar **„Stop“** und entspannen sich komplett. Nach einigen Sekunden werden wir Sie ermuntern, noch einmal Ihre maximale Kraftleistung zu erbringen (wie eben im warm-up). Im Anschluss können Sie sich dann komplett entspannen. Hier werden wir Sie noch zweimal stimulieren: Wenn Sie ihre maximale Leistung erbringen und im Anschluss, wenn Sie sich komplett entspannen.

Abbruchkriterien

- Wenn die rote Linie in einem Durchgang dreimal, für maximal eine Sekunde, unter die schwarze horizontale Linie geht, dann wird der Test abgebrochen.
- Wenn die rote Linie in einem Durchgang einmal, für länger als eine Sekunde, unter die schwarze horizontale Linie geht, dann wird der Test ebenfalls abgebrochen.

Zusammenfassung

Sie bearbeiten die Kraftleistungsaufgabe **so lange Sie möchten**. Für jeden Durchgang bekommen Sie 50 Cent. Sie bekommen **also 50 Cent pro Minute**. Sie bekommen **vorab einen Bonus von XX Euro**. Die Messung geht so lang, **bis Sie aufhören wollen** oder **eines der Abbruchkriterien eintritt**. Wichtig: **Wenn Sie aufhören wollen, dann sagen Sie bitte deutlich vernehmbar „Stop“.**

Haben Sie noch Fragen? Ansonsten kann es losgehen!
